# Bullying victimization among internationally adopted adolescents: Psychosocial adjustment and moderating factors

**DOI:** 10.1371/journal.pone.0262726

**Published:** 2022-02-03

**Authors:** Laura Ferrari, Simona Caravita, Sonia Ranieri, Elena Canzi, Rosa Rosnati

**Affiliations:** 1 Department of Psychology, Family Studies and Research University Centre, Università Cattolica del Sacro Cuore, Milan, Italy; 2 Norwegian Centre for Learning Environment and Behavioural Research in Education, University of Stavanger, Stavanger, Norway; 3 Department of Psychology, Università Cattolica del Sacro Cuore, Brescia, Italy; 4 Department of Psychology, Family Studies and Research University Centre, Università Cattolica del Sacro Cuore, Piacenza, Italy; University of Texas at Dallas, UNITED STATES

## Abstract

Bullying constitutes a serious risk factor for the psychosocial adjustment of young people in both the general population and minority groups. Among minorities, international adoptees are likely to show a specific vulnerability to the experience of being bullied, moderated by specific risk and protective factors. This study aimed to investigate the association between adoptees’ experience of bullying victimization and their psychosocial adjustment, and to explore the moderating role of adoptive identity and reflected minority categorization. An online, anonymous self-report questionnaire was completed by 140 adolescents (13–17 years), who were internationally adopted by Italian families. Findings showed that being victimized was associated with higher levels of emotional and behavioral difficulties, but that the strength of this relation varied according to the levels of adoptive identity and reflected minority categorization. Specifically, victimization was found to have a more detrimental and negative impact on psychological adjustment for adoptees who were highly identified with the adoptive group, and reported to be less perceived by others as members of the minority group. Results are discussed in relation to recommendations for further research as well as for professionals working with internationally adopted adolescents.

## Introduction

Bullying constitutes a particularly serious type of peer victimization and is a risk factor for the psychosocial adjustment of both perpetrators and victims [1,2]. During the past two decades, there has been an increasing corpus of studies focused on the individual and the background characteristics of youth to identify the risk factors that may be precursors to either bullying or victimization. Although the number of studies about bullying is high, and a lot of knowledge has already been gathered, many aspects of bullying need to be further investigated, especially when considering bullying targeted at minority groups [3], who may be at an increased risk of victimization, and who may have limited resources to cope with these events.

When young persons are bullied, specifically because of their belongingness to an ethnic minority group, literature refers to it as *ethnic bullying*. Ethnic bullying sums up distinctive characteristics of general bullying and discrimination [3]. Studies on this topic have shown that youth with minority ethnic background are more likely to experience bullying from their peers [3,4]. The experience of being bullied because of one’s ethnic background is also linked to higher levels of psychological maladjustment (for a review [5]).

Internationally adopted youth represent a unique ethnic minority group, but the risk of peer victimization and the influence on their psychosocial adjustment are almost unexplored, with very few exceptions. Two recent studies carried out in the European context, which is prevalently ethnically homogeneous, are consistent in showing international adoptees’ high vulnerability to bullying [6,7]. Studies carried out in U.S. suggest that internationally adopted children must confront multiple specific challenges related to their adoptive status, their past experience of institutionalization and deprivation, and also their minority ethnic background, with subsequent negative influence on their social competence and peer relationships [8–10]. These factors might expose them to a high risk of peer victimization as well as increase their vulnerability to the experience of being bullied. Nevertheless, these possible associations are still inadequately investigated.

To partially fill this gap, the present study aimed to examine the association between the experience of victimization among internationally adopted adolescents and their psychosocial adjustment, and to explore the risk and protective factors that may have a potentially moderating role. Specifically, to fully understand the nature of the processes that may underlie the vulnerability to peer victimization among international adoptees, we explored whether and to what extent the adoptive identity (how proud the adoptees feel about being adopted) and the *reflected minority categorization* (whether the adoptees believe that others perceive them as belonging to ethnic minority groups) influence the association between victimization and psychosocial outcomes.

### Peer victimization and psychosocial adjustment among adoptees

Research on the general population has found high levels of emotional and behavioral difficulties, including aggression (e.g., [11,12]), greater psychological distress, and lower academic performance (e.g., [13–15]) among youths who are victimized by peers. In fact, peer victimization has been recognized as a relevant social issue in childhood and adolescence, and has been found to be associated with interpersonal problems and internalizing symptoms, including loneliness, depression, and anxiety [2,16–18]. Results of studies carried out both in Europe and U.S. are consistent in showing that being the target of ethnic bullying by peers is associated with psychological maladjustment, especially among adolescents belonging to ethnic minority groups (e.g., [5,19–25]).

However, very limited research is available on ethnic bullying among international adoptees. Two recent studies carried out in Finland and Portugal have indicated that international adoptees are less overtly aggressive towards peers and are less likely to be classified as bullies in comparison to non-adopted peers, but they are more likely to be victimized [6,7]. Moreover, the study carried out by Pitula and colleagues [6] provided evidence for an association between peer victimization and heightened levels of internalizing problems among international adoptees. However, to date, there has been a paucity of studies on this topic, particularly on the possible moderating variables that could weaken or worsen this association.

### Victimization and psychosocial adjustment: The moderating role of adoptive identity

In recent years, an increasing degree of attention has been dedicated to investigating possible protective and risk factors that could play a moderating role in the relationship between peer victimization and psychosocial outcomes, focusing on some dimensions of identity. Specifically, studies on youth minority groups have shown that ethnic identity mitigates the negative effects of ethnic peer discrimination on adolescents’ psychological well-being [26].

However, considering adoptees, a few studies have explored whether ethnic identity could moderate the association between peer victimization and psychological adjustment, and the results have not been consistent. For instance, ethnic identity turned out to play an exacerbating role among adopted Korean American adolescents [27] and a weakening one when considering Latino adolescents adopted in Italy [28]. Fundamentally, adoptees must cope with their distinctive individuality that increases the complexity of their identity development [29]. Moreover, ethnic identity is one of the most crucial dimensions of social identity among adoptees; however, this dimension is strictly intertwined with their adoptive status, as they belong to an ethnic minority because of their birth origin, whilst also to the majority group because of their adoptive status. These findings are consistent in both ethnically more homogenous (Italy) and heterogenous (U.S.) contexts [30,31].

In recent years, considerable research has specifically focused on this significant identity dimension, which is especially relevant to adoptees, that is adoptive identity or an understanding of what it means to be an adopted person. It refers to a sense of belonging and identification, together with commitment, pride, and positive feelings about this identity [32–35]. The adoptive identity refers to the adoptees’ ability to reflect on the meaning of their connection to both their adoptive and birth family, thus making sense of adoption in their life story. Thus, coherent identity formation means revisiting one’s adoption narrative, integrating the adoptee status into one’s sense of self, making sense of looking dissimilar to one’s adoptive family, reconciling differences that might exist between one’s birth and adoptive families, and coming to terms with the circumstances surrounding one’s adoption. Studies suggest that adoptive identity is related to healthier relationships, higher levels of self-esteem, positive affect about adoption, and fewer internalizing problems [33,36–38].

Despite the relevance of adoptive group membership to the psychosocial adjustment and social stigma stemming from adoptive status [39], there is a lack of research exploring the moderating role of adoptive identity in the association between peer victimization and psychological adjustment. We can suppose that adopted adolescents with higher levels of coherent adoptive identity may report more emotional and behavioral problems caused by bullying victimization. This may happen because these discriminatory events are likely to directly harm their feelings of pride about being adopted (i.e., adoptive identity), and thus profoundly hurting them and negatively affecting their adjustment.

### Victimization and psychosocial adjustment: The moderating role of reflected minority categorization

As mentioned above, empirical studies carried out in several countries are consistent in showing that youths with minority ethnic background are more likely to experience ethnic bullying from their peers [3,4,26]. There is also evidence that international adoptees endure a distinctive, contradictory, and unique ethnic experience, called the *adoption paradox* [31], as they are members of the majority culture due to adoption, but at the same time they belong to the ethnic minorities because they are born and have lived mostly for a long period in a foreign country. This implies that they do not share similarities in physical appearance with their own adoptive parents, and are often different from most of their peers and significant others too. Moreover, such ethnic differences can reveal one’s history of adoption to others. Therefore, a peculiar type of social disclosure of adoption occurs: the sharing of adoption with other people, including peers, with whom the adoptee and other family members interact [40], not chosen by themselves, but driven by the physical differences. In cases of same-race placements, adoptees may be considered more phenotypically similar to the majority group, thus they are more likely to be categorized by others as members of the majority group. This process differs for adoptees with transracial placements, as the ethnic difference can lead them to be categorized by others as ethnic minorities, with the same social categorization process applied to migrants, increasing the adoptees’ risk of being victimized in ethnic bullying situations. Evidence from Italy suggests that international adoptees with transracial placements are highly vulnerable to the experience of discrimination in ethnically homogeneous countries [28,41], as is the case with immigrant ethnic minority peers, as reported in a number of countries [42,43].

In line with Cooley’s [44] concept of *looking-glass self*, suggesting that individuals think of themselves based on how they believe others think of them, some studies have revealed the relevance of the perception of other’s appraisal (named as reflected appraisal) in defining and shaping one’s identity in terms of ethnic categorization, especially in the case of multiracial individuals [45,46]. However, there is a lack of research exploring how adoptees believe that others perceive themselves from the point of view of ethnic minority categorization, and whether this reflected appraisal can affect the association between discrimination and psychosocial adjustment. The reflected minority categorization (i.e., others ascribing adoptees as members of ethnic minorities according to their appearance and somatic traits) may influence the association between being peer-victimized and adjustment outcomes in the case of international adoptees. Specifically, we can hypothesize that adoptees who perceive themselves as being categorized by others as a member of an ethnic minority group are more likely to attribute the experience of being bullied by peers to the perpetrator’s racism (external attribution). Conversely, adoptees who perceive themselves as categorized by others into the majority group might more easily attribute the experience of being bullied to their own behaviors and individual traits. This latter dispositional attribution is likely to increase their suffering and consequent maladjustment.

### The current study

**Fig 1:**
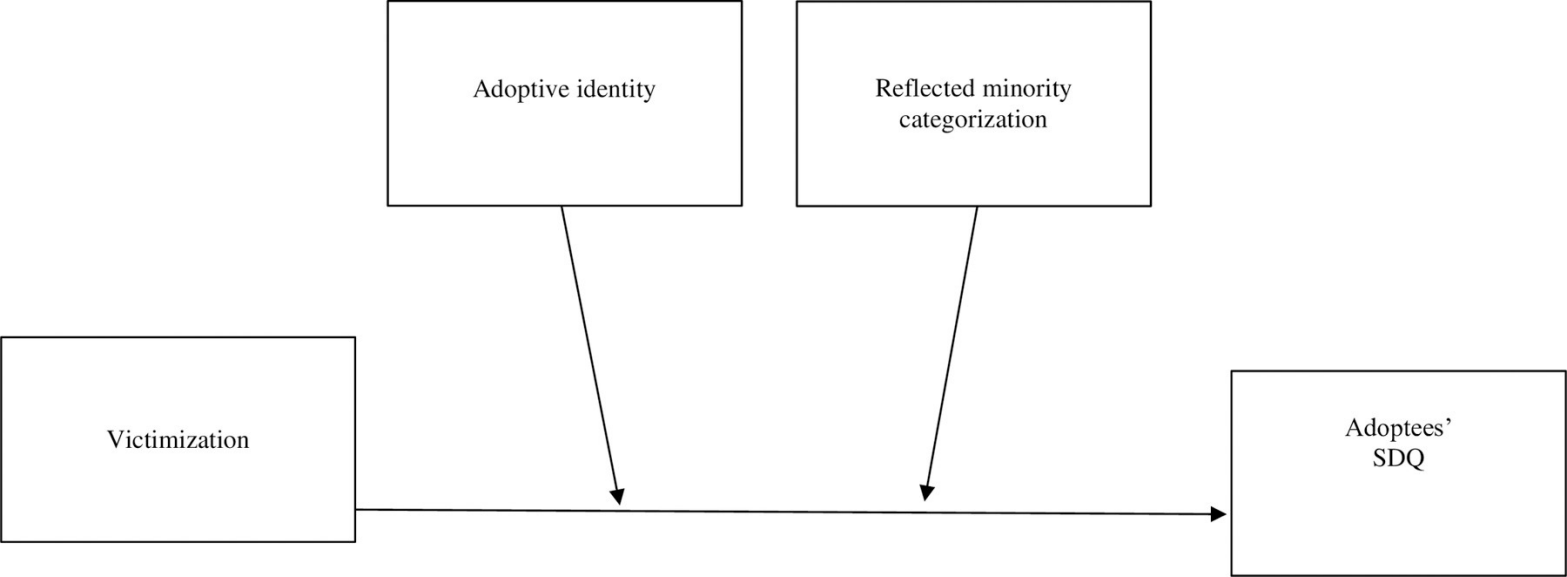
Theoretical moderation model.

The current study focused on exploring, among internationally adopted adolescents, the association between peer victimization in bullying and psychosocial adjustment. In addition, it examines the moderating effects of adoptive identity and reflected minority categorization in this association. To the best of our knowledge, it is the first study that analyzes the potential moderating effects of adoptive identity and reflected minority categorization. In particular, adoptive identity and reflected minority categorization were examined as moderators in the link between being bullied and adoptees’ psychosocial adjustment in terms of emotional and behavioral difficulties (Fig 1).

Based on literature review, we aimed at examining the following hypotheses.

Hypothesis 1: Being victimized in bullying situations will be associated with emotional and behavioral difficulties among adopted adolescents.

Hypothesis 2: The association between victimization in bullying situations and emotional and behavioral difficulties would be weaker for the adoptees with lower levels of adoptive identity, whereas the negative influence of being victimized would be stronger for the adolescents with higher levels of adoptive identity. We hypothesize that being victimized can be particularly harmful for adolescents with high levels of adoptive identity because the victimization suffered by adoptees often targets their adoptive status, thus increasing the hardship of this experience for those who strongly identify with this status, and thus hindering their personal adjustment.

Hypothesis 3: The link between victimization and emotional and behavioral difficulties may be stronger among adolescents with a low level of reflected minority categorization, as these adolescents are more likely to attribute the reasons of being victimized to their own personal characteristics. Among the adoptees who have a high level of reflected minority categorization, victimization may be associated with maladjustment at a lower extent, as they may attribute the cause of bullying to their belonging to a minority ethnic group.

## Methods

### Participants and procedure

The participants comprised 140 adolescents (53.6% males, 46.4% females) who were internationally adopted by Italian families. They were on average 14.97 years old (*SD* = 1.40; range 13–17) with an average age at placement of 5.43 years (*SD* = 3.41; range 0–15). Regarding their country of origin, the sample was distributed as follows: Eastern Europe (35.7%), Asia (35.7%), Latin America (22.9%), and Africa (5.7%). Most of the participants’ adoptive parents (92.1%) were married; 97.1% of the adoptees lived with both adoptive parents, while 2.9% lived only with the adoptive mother. Regarding the place of residence, 70.5% of the participants were from North Italy, 23.7% from Central Italy, and 5.8% lived in South Italy or on an island.

The adoptees were recruited through the collaboration of adoption agencies and adoption professionals located all over Italy and working in the field of international adoption. The agencies contacted the adoptive parents by email, explaining the general aim of the project and asking them to take part in the study. The study was approved by the Ethics Committee of the Department of Psychology of the Università Cattolica del Sacro Cuore (code 15–17), and the study was conducted in accordance with the APA ethical guidelines for human research (http://www.apa.org/ethics/code/). Written informed consent of parents was obtained for all the participants prior to their participation. The adolescents whose adoptive families consented were informed about the main objectives of the study, and they were asked to fill out an online, anonymous self-report questionnaire.

### Measures

Participants completed a self-reported questionnaire containing questions about their socio-demographic characteristics and measures regarding the constructs of interest.

#### Victimization in bullying episodes

Participants filled out seven items from the revised Olweus Bully/Victim Questionnaire [47] to measure the frequency they had experienced each of the bullying episodes on a scale from 1 to 5 (1 = Did not occur; 2 = Occurred once or twice; 3 = Occurred two or three times a month; 4 = Occurred about once a week; 5 = Occurred several times a week). The measure includes items such as: “Other students left me out of things on purpose, excluded me from their group of friends, or completely ignored me;” and “I was called mean names, was made fun of, or teased in a hurtful way.” To code victimization, both categorical and dimensional approaches were used. In the categorical approach, individuals were classified as “chronic” victims if they exceeded a cut-off of two or three episodes a month as victims in the last two months, or “occasional” victims, based upon a response of “once or twice” in the last two months. In the dimensional approach, a total score of victimization was computed by averaging the items. A higher score indicates a greater frequency of victimization. Cronbach’s Alpha: .78.

#### Adoptive identity

To measure adolescents’ adoptive identity, we used an adapted version of the Multigroup Ethnic Identity Measure [48]. Specifically, this scale is composed of four items on a 4-point response scale (from 1 = Never to 4 = Always), and it is aimed at gauging positive feelings related to being adopted. The total score was created by averaging the items. An item example is: “When I think of being adopted, I feel proud.” A higher score indicates a higher level of adoptive identity. The Cronbach’s alpha showed a .66 satisfactory internal consistency.

#### Reflected minority categorization

We used an ad hoc one-item measure with a 4-point response scale (from 1 = Never to 4 = Often): “If a stranger meets me, he/she thinks I am a foreigner rather than an Italian.”

#### Emotional and behavioral difficulties

The presence of emotional and behavioral difficulties was measured by administering the Strengths and Difficulties Questionnaire (SDQ) developed by Goodman [49]. This scale consists of 20 items measured on a scale ranging from 0 (Not true) to 2 (Absolutely true) assessing the frequency with which emotional and behavioral difficulties were experienced within the past three months (e.g., “I get a lot of headaches, stomach-aches, or illnesses;” “I am restless; I cannot stay still for long;” and “I am usually on my own; I generally play alone or keep to myself”). Following the procedure by Goodman, Meltzer, and Bailey [50], scores on the single items were summed up, so that the scores on the final total index (alpha = .83) ranged from 0 to 40. Higher scores indicate more emotional and behavioral difficulties. Based on the scoring guidelines (see http://www.sdqinfo.org), for the parent-version, a total score for SDQ falling in the 0–13 range is considered “normal” (clinically significant problems are unlikely to occur), a score of 14–16 as “borderline” (slight risk of clinically significant problems), and a score of 17–40 as “clinical” (substantial risk of clinically significant problems).

### Data analyses

Descriptive statistics were computed for all the variables. A univariate analysis of variance (ANOVA) was carried out to investigate possible differences in bullying victimization, adoptive identity, reflected minority categorization, and emotional and behavioral difficulties depending on adolescents’ gender (0 = male, 1 = female) and current age (2 levels: children below 15, adolescents aged between 16 and 17). Pearson’s correlations coefficients were used to investigate the associations between the variables.

To examine the moderating effects of adoptive identity and reflected minority categorization (moderators) in the link between victimization (independent variables) and adoptees’ psychosocial adjustment (dependent variable), we used the Process Macro for SPSS [51], applying Model 2 with 5000 bias-corrected bootstrap samples. Regression analyses were conducted in which coefficients were bootstrapped using 5,000 bootstrap samples. The coefficients were tested for statistical significance by means of the percentile confidence intervals, and a significant effect is said to occur if the 95% confidence interval excluded 0. According to Aiken, West, and Reno [52], in simple slope analyses, the function was graphed for two levels of independent variable and moderators—1 *SD* above the mean and 1 *SD* below the mean.

## Results

Descriptive statistics and correlations are presented in Table 1. Regarding the categorical coding of victimization, 19.3% of adoptees were classified as “chronic” victims, 31.4% as “occasional” victims, and 49.3% were not victims. Moreover, they reported medium-high levels of adoptive identity and reflected minority categorization. The average level of emotional and behavioral difficulties reported by adoptees was lower than the clinical cut-off score.

**Table 1:** Means (*M*), Standard Deviations (*SD*) and Correlations between the study variables (*N* = 140).

	*M (SD)*	1	2	3	4
**1. Victimization**	1.29 (0.51)	-			
**2. Adoptive identity**	3.04 (0.73)	-.28**	-		
**3. Reflected minority categorization**	2.54 (1.15)	.21*	-.13	-	
**4. Emotional and behavioral Difficulties**	11.38 (6.53)	.43**	-.43**	.11	-

*Note*. The scale ranges from 1 to 5 for perceived victimization; from 1 to 4 for adoptive identity and for reflected minority categorization; from 0 to 40 for emotional and behavioral difficulties (“normal range”: 0–13; “borderline”: 14–16; “clinical score”: 17 to 40).

**p < .01.

*p < .05.

The univariate ANOVA indicated that there were no significant effects of adoptees’ current age on victimization, adoptive identity, reflected minority categorization, and emotional and behavioral difficulties. No significant differences were found related to adoptees’ gender referring to the victimization, adoptive identity, and reflected minority categorization, whereas a significant gender difference emerged referring to the emotional and behavioral difficulties. Specifically, females were more likely to experience more emotional and behavioral difficulties than males [females: *M* = 12.68; *SD* = 6.88; males: *M* = 10.36; *SD* = 6.11; *F*(1, 137) = 4.069, *p* = .05, η^2^ = .03]. No interaction effects were found.

Correlations revealed positive associations between victimization and emotional and behavioral difficulties, and a positive association between victimization and reflected minority categorization; negative correlations were found between adoptive identity and both victimization and emotional and behavioral difficulties. No significant correlations emerged between reflected minority categorization, on the one hand, and emotional and behavioral difficulties and adoptive identity, on the other.

To test the moderating role of adoptive identity and reflected minority categorization in the association between victimization (independent variable—predictor) and emotional and behavioral difficulties (dependent variable—criterion), we conducted hierarchical regression analyses. In the moderation regression model (Table 2), we included the adoptees’ age and gender as control variables. The association between the adoptees’ victimization and their emotional and behavioral difficulties was moderated by both adoptive identity (interaction victimization* adoptive identity: *B* = 2.64, *p* = .01) and reflected minority categorization (interaction victimization* reflected minority categorization: *B* = -1.77, *p* = .05). These significant interactions indicated that the association between victimization and emotional and behavioral difficulties became stronger with an increase in the levels of adoptive identity (positive interaction) and with a decrease in the levels of reflected minority categorization (negative interaction).

**Table 2:** Hierarchical regressions testing the model proposed for adoptees’ emotional and behavioral difficulties (*N* = 140).

	*B*	*t*
**Victimization**	3.61	.83
**Adoptive identity**	-6.21	-3.96***
**Reflected minority categorization**	2.13	1.69
**Age of adoptees**	.39	1.22
**Gender**	2.59	2.85**
**Victimization × Adoptive identity**	2.64	2.53*
**Victimization × Reflected minority categorization**	-1.77	-1.91*
***R*** ^***2***^		0.38
***F***		11.66***
***Df***		(7,132)

*p < .05.

**p < .01.

***p < .001.

**Fig 2:**
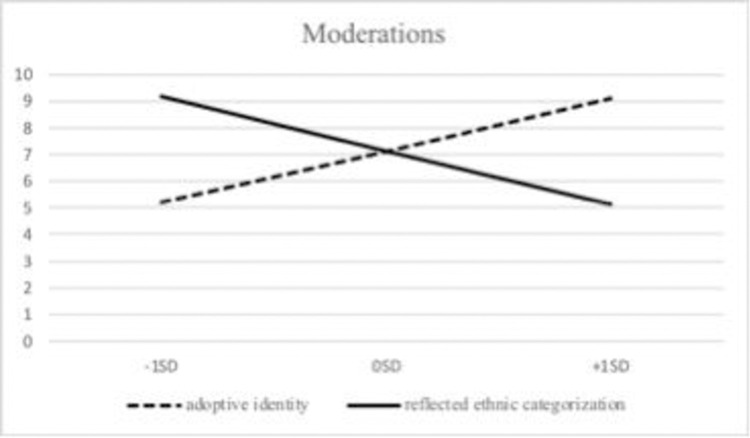
Moderating effect of adoptive identity and reflected minority categorization on the relation between victimization and psychosocial adjustment.

To interpret these interactions more properly, following Aiken, West, and Reno [52], conditional effects were examined. For adolescents with higher adoptive identity (+1 *SD*), the association with being victimized was stronger: *B* = 9.06, *p* < .01; 95% *CI* = [5.48, 12.64], than for those with low adoptive identity (-1 *SD*): *B* = 5.22, *p* < .01; 95% *CI* = [2.37, 8.07]. The association between victimization and emotional and behavioral difficulties was stronger for adoptees who perceived a lower level of reflected minority categorization (-1 *SD*): *B* = 9.17, *p* < .01; 95% *CI* = [5.05, 13.28], whereas this association was lower for those with higher reflected minority categorization (+1 *SD*): *B* = 5.11, *p* < .01; 95% *CI* = [2.61, 7.60]. With reference to adoptive identity and reflected minority categorization, the conditional effect was significant (*p* < .01) for all the levels of the moderators. Fig 2 illustrates the moderation effects.

## Discussion

The current study investigated the relation between international adoptees’ experience of victimization and emotional and behavioral difficulties, as well as tested the moderating role of adoptive identity and reflected minority categorization.

The findings of the correlation analysis showed that being victimized was associated with higher levels of emotional and behavioral difficulties. Literature is consistent in showing that being bullied is related with higher risk of psychological maladjustment both in the general population and in minority groups [5,11]. Our results further extend this association to the unique minority group represented by international adoptees. In agreement with previous European studies suggesting peer problems (e.g., [53–55]) and difficulties in creating and maintaining friendly relationships among adoptees (e.g., for a metanalysis [56]), we could speculate that adoptees might have fewer relational resources to interact with and to protect themselves from discriminatory behaviors. Moreover, our results suggest that internationally adopted adolescents reporting higher feelings of pride for being adopted (i.e., adoptive identity) showed lower levels of emotional and behavioral symptoms. This outcome is in line with previous research indicating that adoptive identity can be a protective factor associated with higher levels of psychosocial adjustment [37,38]. In the analyses of correlation indices, adoptive identity was negatively associated with victimization by peers. This result may be interpreted in light of the fact that being adopted may be one of the dimensions that are targeted in bullying episodes, for instance, by means of verbal aggressions. Hence, adoptive identity may be hindered by being victimized. A positive correlation between levels of bullying victimization and being categorized by others as part of the minority groups also emerged. This result might be explained by the fact that adoptees who are perceived by others as ethnically different are more likely to be targeted because they belong to an ethnic minority group. Even if we focused on general bullying, this interpretation is consistent with studies highlighting that adolescents with an ethnic minority background are more likely to be victims of bullying than their peers both in ethnically more heterogeneous (U.S.) and homogeneous (Italy) contexts [3,42]. Future studies may investigate ethnic bullying among adoptees to better explore this interpretative hypothesis.

The association between frequent episodes of victimization and high levels of behavioral and emotional difficulties turned out to be fully moderated by both adoptive identity and reflected minority categorization, thus confirming our hypotheses concerning the moderating effects.

As expected, the association between peer-victimization and emotional and behavioral difficulties in adoptees was moderated by the strength of their adoptive identity. It turned out that the association between being victimized and higher levels of maladjustment is stronger among those adoptees who have higher adoptive identity. It is possible that the experience of victimization, often related to the adoptive status [57], more strongly affects adolescents who feel proud of their adoptive status and strongly identify with the adoptive group. We could infer that these discriminating events can affect a central and relevant dimension of the adoptees’ identity, thus profoundly hurting them. Indeed, when individuals are highly identified with their in-group (for example, the adoptive group), episodes of group related discrimination are more likely to be appraised as self-relevant. The more the belongingness to and the identification with the group is central and a core aspect of self, the more the discriminatory events are psychologically significant and threatening [58]. Further research should more specifically consider the role of nuanced dimensions of adoptive identity such as centrality.

As far as reflected minority categorization is concerned, this variable also played a moderating role, thus confirming our third hypothesis. The adoptees who perceived others’ appraisal about them as members of the minority group reported lower psychosocial difficulties in association with experiencing victimization. This moderation effect could be considered as the result of a specific attribution process. Adoptees with a stronger reflected minority categorization might consider their own ethnic group as the target of ethnic victimization (external attribution) and not directly themselves as individual targets because of their own characteristics (internal attribution). This process can explain the possible moderation effect played by reflected minority categorization in the association between experiencing peer victimization and adoptees’ psychological adjustment. Indeed, according to the social identity perspective [59], when individuals belong to a minority group, they can interpret the victimization from an out-group due to the stigmatization of their own group (“I belong to a minority group, and my minority group is victimized”), rather than referring to self-referential explanations [60–62]. As a form of self-serving bias, this external attribution might protect adoptees from the negative consequences associated with being bullied by peers.

The present findings should be considered in light of some limitations. First, our sample size was small, so generalizability of our findings need to be further tested. Second, we focused only on adopted adolescents, who may feel the unique experiences of victimization. Thus, a future research path might be comparing internationally adopted adolescents with other non-adopted adolescents to understand if the former group perceives peer victimization and emotional and behavioral difficulties similarly or differently than the latter. Moreover, our data were driven from adolescents’ self-report measures responded by adolescents and relied on a single-item scale. A multi-method and a multi-item approach are needed to reduce the effect of self-report biases. Interestingly, as individual perceptions of peer victimization elucidate how the respondents actually feel regardless of their interaction with peers, subjective assessment of peer victimization can prove to be a strength. However, in order to have a complete portrait of their experiences and the investigation process complexity, further research could apply other measures, based on other sources of information, such as parents’ or teachers’ reports, to deeper understand factors that may support the adoptees in facing their developmental challenges and in achieving positive and adaptive outcomes. Finally, the data were cross-sectional; therefore, causal inferences cannot be made. Longitudinal studies would provide more accurate statements regarding the direction of the associations between the constructs considered in this study.

Notwithstanding these limitations, the findings from this study have important implications for researchers and professionals working on post-adoption intervention with internationally adopted adolescents. This study provides evidence that adoptees’ experience of peer victimization needs to be considered to identify more appropriate and adequate interventions in supporting their psychosocial adjustment. Specific programs should be designed to help adoptees cope with episodes of victimization, considering that the associations between being victimized and emotional and behavioral difficulties vary as a function of adoptive identity and adoptees’ perception of others’ appraisal of them as members of an ethnic minority group. In this line, this study provides a first insight into the complex interplay of risk and protective factors in the experience of adoptees’ victimization.
